# Shared Care for Adults with Sickle Cell Disease: An Analysis of Care from Eight Health Systems

**DOI:** 10.3390/jcm8081154

**Published:** 2019-08-02

**Authors:** Arch G. Mainous, Benjamin Rooks, Rebecca J. Tanner, Peter J. Carek, Vandy Black, Thomas D. Coates

**Affiliations:** 1 Department of Health Services Research Management, and Policy, University of Florida, Gainesville, FL 32610, USA; 2 Department of Community Health and Family Medicine, University of Florida, Gainesville, FL 32610, USA; 3 Division of Hematology and Oncology, Departments of Pediatrics, University of Florida, Gainesville, FL 32610, USA; 4 Department of Pediatrics and Pathology, University of Southern California Keck School of Medicine, Los Angeles, CA 90027, USA

**Keywords:** sickle cell disease, shared care, primary care, electronic medical record, secondary data analysis

## Abstract

Adult sickle cell disease (SCD) patients frequently transition from pediatric hematology to adult primary care. We examined healthcare utilization for adult patients with SCD with shared care between hematologists and primary care providers (PCP). We analyzed the OneFlorida Data Trust, a centralized data repository of electronic medical record (EMR) data from eight different health systems in Florida. The number of included adults with SCD was 1147. We examined frequent hospitalizations and emergency department (ED) visits by whether the patient had shared care or single specialty care alone. Most patients were seen by a PCP only (30.4%), followed by both PCP and hematologist (27.5%), neither PCP nor hematologist (23.3%), and hematologist only (18.7%). For patients with shared care versus single specialist care other than hematologist, the shared care group had a lower likelihood of frequent hospitalizations (OR 0.63; 95% CI 0.43–0.90). Similarly, when compared to care from a hematologist only, the shared care group had a lower likelihood of frequent hospitalizations (OR 0.67; 95% CI 0.47–0.95). There was no significant relationship between shared care and ED use. When patients with SCD have both a PCP and hematologist involved in their care there is a benefit in decreased hospitalizations.

## 1. Introduction

Among pediatric patients with sickle cell disease (SCD), survival rates and life expectancies continue to improve [1]. Some recent evidence suggests an approximate survival rate of 95% among patients with SCD up to 18 years of age [1]. The median age at death is approximately 40 years old [2]. However, once patients with SCD become adults the improvements in survival become less evident [3]. Mortality is not the only issue, as surviving adult patients with SCD have renal and pulmonary failure, strokes, and organic brain disease [4,5]. Moreover, morbidity and corresponding health care utilization for emergency departments, hospitalizations, and readmissions are high for young adults with SCD [6,7,8]. This is a rare disease and one that has historically been managed in pediatrics due to the limited life expectancy of patients with SCD.

Most SCD patients transition from pediatrics, particularly pediatric hematology, to adult primary care [9]. Only a minority of adult patients with SCD receive specialist care at a comprehensive SCD treatment center, others may seek care through their primary care provider or through the emergency department. Management of this condition is not similar across settings. There is some indication that primary care providers are not comfortable managing adult patients with SCD [10,11]. Because of the previously limited occurrence of these patients in primary care, providers of adult primary care may not have enough education, experience, or confidence to appropriately care for these patients on their own, thereby bringing up the logistical issues involved in providing a full scope of care in coordination with other providers [12,13]. Furthermore, as these patients age, their medical requirements extend beyond the care related directly to their SCD, resulting in a need for a primary care provider who can manage the complications that are common for all aging adults. Patients with SCD have expressed an understanding of the importance of the role primary care plays in providing for their continual care and a desire to utilize primary care for acute issues [14]. 

A shared care model is one where the primary care provider has a patient who needs both primary care and very specialized medical care. This model has been viewed favorably among primary care providers and has been shown to capture comorbidities and prevent complications to help improve patient outcomes [15]. Several studies have explored the shared care model with patients with a chronic disease who are transitioning from pediatric to adult care. Most studies demonstrating the success of shared care were among patients with cancer, chronic kidney disease, and mental health [16,17,18]. Some studies have indicated a need for further exploration and utilization of the shared care model for patients with SCD [19,20]. 

The purpose of this study was to examine health care utilization for adult patients with SCD using the shared care model between hematologists and primary care providers versus patients with only primary care or only specialty care.

## 2. Experimental Section

The OneFlorida Data Trust is a centralized data repository that brings together electronic medical record (EMR) and health insurance claims data for patients who were seen at the following health care centers or insured by the following providers: Bond Community Health Centers, Florida Hospital, Nicklaus Children’s Hospital, Orlando Health, Tallahassee Memorial HealthCare, UF Health, University of Miami Health, and Health Choice Network. Only EMR data were used for the study. Inpatient, outpatient, and emergency department (ED) records were available in the OneFlorida Data Trust. The data included information regarding providers, patient demographics, encounters, procedures, and diagnoses to provide granular detail about how care was delivered throughout the state of Florida. The data that is collected through the OneFlorida Data Trust is provided to users in a de-identified way. The study was approved by the Institutional Review Board of the University of Florida.

### 2.1. Cohort Definition

Patients were considered to have SCD if they had one or more documented health care encounters with an SCD International Classification of Diseases (ICD) diagnosis code (Table 1) between April 2011 and March 2018. To be included in the study, patients must have had at least 12 continuous months of encounter data after the first SCD diagnosis code appeared in the EMR. The most recent 12 continuous months of data for each patient were used. Because of the importance of the shared care variable and its reliance on ambulatory care, to be included in the sample, patients must have had at least one ambulatory care visit. Only patients aged 21 or older with SCD as of their first healthcare encounter in our dataset were used for this analysis (*n =* 1147). 

### 2.2. Utilization of Healthcare

Emergency department (ED) encounters were defined as visits to the ED resulting in a discharge. Hospital encounters were defined as inpatient hospital stays, ED to inpatient visits, or non-acute institutional stays. Ambulatory care encounters were defined as either ambulatory visits or “other ambulatory visits.” An ambulatory visit was defined as any visit to an outpatient clinic or encounter in a surgical center, urgent care center, or hospital resulting in a same-day discharge, excluding emergency room encounters. “Other ambulatory encounter” was defined as non-overnight hospice, home health, nursing, and other non-hospital visits. Readmissions were defined as an encounter (ED, hospital, or ambulatory care) that occurred within a 30-day window of discharge from a facility of the same type. 

### 2.3. Healthcare Provider Utilization

Physician National Provider Index (NPI) numbers were provided by the OneFlorida Data Trust for a portion of providers. NPI is a provider-specific identifier and the OneFlorida Data Trust provided this information as a way to link specific providers to encounters. Primary care providers (PCPs) were identified by the NPIs for the following provider types: Nurse Practitioner, Nurse Practitioner Adult Health, Nurse Practitioner Primary Care, Nurse Practitioner Obstetrics and Gynecology, Nurse Practitioner Gerontology, Nurse Practitioner Women’s Health, Nurse Practitioner Family, General Practice, Family Medicine, Family Medicine Adult Medicine, Family Medicine Adolescent Medicine, Family Medicine Geriatric Medicine, Internal Medicine, and Clinic/Center Primary Care. Hematologists, pediatricians, and emergency care specialists were likewise identified by their NPI number. If none of the providers a patient saw had an available NPI, that patient was excluded from the analysis. Patients with shared care were defined as those who had one or more encounters with a PCP and one or more encounters with a hematologist in an ambulatory care setting in their most recent continuous 12 months of data.

### 2.4. Frequent Utilizer of Care

Definitions for frequent utilizers of care were set by determining the 90^th^ percentile of visits per encounter type in the data. Patients who visited the ED four times or more were admitted to the hospital more than once, or visited an ambulatory care setting more than 19 times in a year were considered to be frequent utilizers. 

### 2.5. Data Analysis

Associations between provider type in past 12 months (PCP only, hematologist only, both PCP and hematologist, neither) and ED and hospital utilization were assessed using logistic regression. Statistical significance was specified at *p* ≤ 0.05. Estimates of the bivariate odds and rate ratios from each model are reported alongside 95% confidence intervals. All data processing and analysis was performed in the R computing language, version 3.5.2.

## 3. Results

There was a total of 6001 patients, both children and adults, with SCD in our One Florida dataset. Since our study focused on adult patients, the total was reduced to 4489. After exclusions, the number of included adults was 1147. Patients were most commonly seen by only a PCP in the evaluated year (30.4%), followed by both PCP and hematologist (27.5%), neither PCP nor hematologist (23.3%), and hematologist only group (18.7%) (Table 2). Patients who saw neither a PCP nor a hematologist saw a variety of medical practitioners, including surgeons, internal medicine specialists, psychiatrists, ophthalmologists, obstetricians/gynecologists, etc. The proportion of patients who received transfusions in the year under study was 7.9%, and the proportion receiving a prescription for hydroxyurea was 21.7%.

Table 3 shows the results of the logistic regression analyses assessing the impact of healthcare provider usage on being a frequent ED utilizer and a frequent inpatient hospital utilizer. Individuals who saw both a hematologist and a PCP had similar odds of being frequent utilizers of the ED as individuals who saw only a hematologist, only a PCP, or neither. However, individuals with shared care had lower odds of being frequent inpatient hospital utilizers than individuals who saw neither type of provider or a hematologist only.

Although the cohort was limited to patients who most closely fit our conceptual definition, a sensitivity analysis showed that our conclusions were robust when we added back in patients who were left out due to our exclusion criteria. The results were robust with a definition of the cohort including patients with zero ambulatory care visits. 

## 4. Discussion

The results of this study are suggestive of a benefit to a shared care model for adult SCD patients. Patients who sought care from both a PCP and a hematologist during a given 12-month period, were less likely to have a high number of inpatient hospital admissions than patients who sought care from a PCP only, a hematologist only, or some other type of care provider. Although this evaluation was not of a formally structured health care delivery model, the results support the idea of a potential value to implementing a model linking primary care and hematology for adult patients with SCD who are transitioning from or have transitioned from pediatric care. Because SCD is a rare disease, most PCPs have very limited experience caring for SCD patients; thus, a shared care model might better address these patients’ total healthcare needs, with PCPs and hematologists playing complementary roles. 

Patients in this study utilized several types of providers. Some patients with rare diseases may be routinely cared for by their PCP and utilize a specialist on an infrequent, as-needed basis. As children with SCD transition to adult care, it can often be challenging to identify specialists with expertise in SCD care. As individuals with SCD live longer, it is likely that the need for collaborative care will increase. Nearly a quarter of the patients in our sample were 50 years of age or older, which is much higher than reported in prior studies of individuals from earlier birth cohorts [6,21,22]. 

In contrast to the findings for hospital inpatient admissions, in this study, shared care was not associated with ED utilization. It is plausible that shared care is most applicable to buffering more severe episodes of care. The patients with the most complex illness are the ones with shared care.

Another issue that may ultimately impact the delivery of care for SCD patients and hospitalizations is potential discrimination [23]. SCD patient perceptions of discriminatory experiences with healthcare providers are associated with adherence to treatment recommendations. Having a positive relationship between the providers caring for the patient may improve the trust that SCD patients have in their providers. 

A future direction could be to associate adult SCD patient management outcomes with PCP and hematologist involvement in a way documenting whether the providers have an explicit cooperative agreement to share the care for a patient. Analyses may provide a platform to identify and address the need for specialists for adult SCD patients with an additional awareness for PCPs to become trained with general management scenarios for less complex SCD indications.

This study has some limitations. First, the data only pertains to care that occurs within the health systems included in the OneFlorida Data Trust dataset. It does not include care that occurs outside of those health systems; therefore, some medical care that patients received may not be included in the data. Specifically, the receipt of transfusions may be an undercount because of patients receiving transfusions in other settings than the health system (e.g., blood bank). The current study was based on EMR data rather than claims data, and therefore some patients may have out-of-health-center utilization that would not get captured in a health system EHR. Second, patients whose providers did not have an NPI were excluded from analysis. This may have affected shared care versus individual care estimates. Third, this study only evaluated 12 months of utilization. A longer period of study could yield very different results. Since the identification of patients was based on ICD coding, there is the possibility of misclassification. Patients were included in this analysis if they had at least one diagnosis with an eligible ICD-9 or ICD-10 code. Other studies have utilized more stringent definitions; however, they were not using EMR data. Similarly, we did not attempt to define the type of SCD in this study, as prior findings show a poor correlation between ICD coding and sickle cell genotype [24]. However, since sickle cell genotype is a consistent predictor of utilization. Such information will need further exploration in future studies. 

Although the study was not designed to be representative of the state of Florida, the OneFlorida Data Trust EMR participating health systems cover more than two million patients. The EMR consortium is missing health systems in the southwest portion of the state. Finally, the operationalization of shared care is based on multiple providers seeing the same patient as documented in their shared EMR. Our data cannot define exactly how much of an acknowledged shared relationship existed between the PCP and the hematologist in the care of each patient. We feel that there is some implicit relationship/cooperation between the providers because they are in the same health system using the same EMR to care for the same patient. However, the next step to understand that relationship would require gathering the perspective of the two providers on the relationship.

## 5. Conclusions

It appears that frequent inpatient utilization decreased when patients with SCD have a primary care provider and a hematologist involved in their care and that the shared care model is superior to a hematologist alone for decreasing the likelihood of hospitalizations. More research is needed to see if a shared care model can be used as an intervention to decrease SCD hospitalizations. 

## Figures and Tables

**Table 1:** ICD Codes used to Identify Patients with Sickle Cell Disease.

	Hemoglobin SS	Thalassemia	Hemoglobin SC	Other Sickle Cell
Without Crisis	282.61	282.41;D57.4	282.63;D57.2	282.6;282.68;D57; D57.1;D57.8
With Crisis	282.62;D57.0, D57.01;D57.02	282.42;D57.41; D57.411;D57.412; D57.419	282.64;D57.21	282.69;D57.81; D57.811;D57.812; D57.819

**Table 2:** Characteristics of the patients with sickle cell disease (SCD) in the One Florida Data Trust dataset.

	Count (N)	Prevalence
Female	707	61.72%
Black	987	86.1%
White	156	13.6%
Other	4	0.3%
Age		
21–34	557	48.6 %
35–49	313	27.3%
50+	277	24.1%
ED Visits		
0	859	74.9%
1+	288	25.1%
Frequent ED Utilizers *	102	8.9%
Hospital Visits		
0	877	76.5%
1+	270	23.5%
Frequent Hospital Utilizers *	153	13.3%
Ambulatory Visits		
1–5	473	41.2%
6+	674	58.8%
Frequent Ambulatory Care Utilizers *	199	17.4%
Healthcare Provider Type		
Hematologist and PCP	316	27.5%
Hematologist	215	18.7%
PCP	349	30.4%
Neither PCP nor Hematologist	267	23.3%

* Patients who visited the emergency department (ED) four times or more, were admitted to the hospital more than once, or visited an ambulatory care setting more than 19 times in a year were considered to be frequent utilizers.

**Table 3:** Odds Ratios for Utilization and Shared Care Status.

	Frequent ED Utilizer Odds Ratio (95% CI)	Frequent Inpatient Hospital Utilizer Odds Ratio (95% CI)
Hematologist and PCP vs. Neither	0.96 (0.62, 1.49)	0.63 (0.43, 0.90)
Hematologist and PCP vs. Only Hematologist	1.10 (0.72, 1.65)	0.67 (0.47, 0.95)
Hematologist and PCP vs. Only PCP	0.94 (0.62, 1.40)	0.75 (0.53, 1.04)
